# Systematic Identification of Intracellular-Translocated Candidate Effectors in *Edwardsiella piscicida*

**DOI:** 10.3389/fcimb.2018.00037

**Published:** 2018-02-16

**Authors:** Lingzhi Zhang, Zhiwei Jiang, Shan Fang, Yajun Huang, Dahai Yang, Qiyao Wang, Yuanxing Zhang, Qin Liu

**Affiliations:** ^1^State Key Laboratory of Bioreactor Engineering, East China University of Science and Technology, Shanghai, China; ^2^Shanghai Engineering Research Center of Maricultured Animal Vaccines, Shanghai, China; ^3^Shanghai Collaborative Innovation Center for Bio-Manufacturing Technology, Shanghai, China; ^4^Laboratory for Marine Biology and Biotechnology, Qingdao National Laboratory for Marine Science and Technology, Qingdao, China

**Keywords:** effector, *Edwardsiella piscicida*, T3SS, T6SS, OMV

## Abstract

Many bacterial pathogens inject effectors directly into host cells to target a variety of host cellular processes and promote bacterial dissemination and survival. Identifying the bacterial effectors and elucidating their functions are central to understanding the molecular pathogenesis of these pathogens. *Edwardsiella piscicida* is a pathogen with a wide host range, and very few of its effectors have been identified to date. Here, based on the genes significantly regulated by macrophage infection, we identified 25 intracellular translocation-positive candidate effectors, including all five previously reported effectors, namely EseG, EseJ, EseH, EseK, and EvpP. A subsequent secretion analysis revealed diverse secretion patterns for the 25 effector candidates, suggesting that multiple transport pathways were involved in the internalization of these candidate effectors. Further, we identified two novel type VI secretion system (T6SS) putative effectors and three outer membrane vesicles (OMV)-dependent putative effectors among the candidate effectors described above, and further analyzed their contribution to bacterial virulence in a zebrafish model. This work demonstrates an effective approach for screening bacterial effectors and expands the effectors repertoire in *E. piscicida*.

## Introduction

Bacterial pathogens utilize a multitude of methods to invade mammalian hosts, damage tissue sites, and thwart the immune system from responding. One essential component of these strategies for many bacterial pathogens is the secretion of proteins across phospholipid membranes. Secreted proteins can play many roles in promoting bacterial virulence, from enhancing attachment to eukaryotic cells, to scavenging resources in an environmental niche, to directly intoxicating target cells and disrupting their functions. Bacterial pathogens often transfer an arsenal of virulence factors, referred to as effectors, into host cells during infection (Christie et al., 2005; Galán et al., 2014; Ho et al., 2014). Identification of functional bacterial effectors is critical to our understanding of pathogen-host interactions and bacterial pathogenesis. Although the protein components of bacterial secretion machines are highly conserved, the sequences of effector proteins are not homologous between bacterial species, indicating plasticity in the repertoire of the effectors (Galán, 2009; Dean, 2011). Experimental identification of novel effectors relies on translocation assays using fusion proteins of a putative effector with a reporter gene. Before testing putative effectors in an appropriate assay, genes encoding these proteins must be extracted from the full genome.

Many sequence-based bioinformatics tools have been developed to predict putative effectors across bacterial genera, usually based on the similarity to known effectors, the presence of secretion/translocation signals and specific domains and motifs, or more comprehensive machine learning algorithms (Arnold et al., 2009; Burstein et al., 2009; Samudrala et al., 2009; Buchko et al., 2010; McDermott et al., 2011). In addition, some other biochemical and genetic methods have been used to screen bacterial effectors, including secretion proteomics (Guttman et al., 2002; Deng et al., 2010), transcriptional regulation (Schechter et al., 2006), and chaperone-binding methods (Costa et al., 2012). Although these approaches have provided information about some bacteria, it is still necessary to develop complimentary screening strategy to elucidate the full repertoire of effectors in bacteria.

*Edwardsiella piscicida* (*E. piscicida*) is a pathogenic bacterium that infects a wide range of hosts, from fish to birds, reptiles, and humans (Mohanty and Sahoo, 2007; Leung et al., 2012). Previous studies have shown that T3SS and T6SS mechanisms are essential for the virulence of *E. piscicida* (Rao et al., 2004; Tan et al., 2005; Zheng and Leung, 2007; Wang et al., 2009; Yang et al., 2012); however, functional effectors in *E. piscicida* are largely unknown. In recent years, only four T3SS effectors, EseG (Xie et al., 2010), EseH (Hou et al., 2017), EseJ (Xie et al., 2015), EseK (Cao et al., 2017), and a T6SS effector, EvpP (Chen et al., 2017), have been found to play important roles in bacterial infections. Because bacterial effectors are essential to hijacking the host cellular machinery to create a suitable niche for bacterial survival and proliferation, we assume that the bacterial effectors might be transcriptionally regulated by intracellular infection signals. Therefore, infection-responsive bacterial genes might be the key to screening for functional effectors.

In our previous work, we established a macrophage infection model for *E. piscicida*, in which a population of intracellularly-replicating *E. piscicida* was released into the supernatant through induction of a caspase-1-dependent cell pyroptosis at a late phase of infection and exhibited a T3SS/T6SS-responsive transcription profile (Zhang et al., 2016). Based on the infection-responsive genes identified, the top 200 regulated genes from over 3,000 genes were subsequently tested in a translocation assay in this work, and a total of 25 intracellularly translocation-positive candidate effectors were verified, among which five were previously identified and the other 20 are first reported here. We also verified two new T6SS-dependent putative effectors and three outer membrane vesicle (OMV)-dependent putative effectors from the candidates and assessed their importance to *E. piscicida* virulence in zebrafish. Our results expanded the known effector repertoire of *E. piscicida* and increase our understanding of *E. piscicida* virulence mechanisms.

## Materials and methods

### Bacterial strains and cell culture

Wild type *E. piscicida* EIB202, the T3SS mutant (ΔT3SS), and the T6SS mutant (ΔT6SS) have been described previously (Yang et al., 2015). *Escherichia coli* DH5α, cc118 λ*pir*, and SM10 λ*pir* were used as hosts for the construction of plasmids and for the conjugation of the *pir*-dependent suicide plasmid. The bacterial strains used and constructed in this study are listed in Table S2. *Escherichia coli* were grown in Luria-Bertani (LB) broth or on LB agar at 37°C, while *E. piscicida* strains were grown in Trypticase Soy Broth (TSB), Dulbecco's modified essential medium (DMEM) or on tryptic soy agar (TSA) at 30°C. HeLa cells (ATCC number CCL-2) were cultured at 37°C in a 5% CO_2_ atmosphere in a growth medium (DMEM supplemented with 10% fetal bovine serum [FBS]).

### Construction of plasmids and effector-deletion mutants

To construct the *E. piscicida* specific gene deletion strain, in-frame deletion mutants of the effector genes were generated by the *sacB*-based allelic exchange as previously described (Yang et al., 2015). The fragments upstream and downstream of each effector gene were fused by overlapping PCR. These fragments were then cloned into the *sacB* suicide vector pDMK and linearized with *Bgl*II and *Sph*I, and the resulting plasmids were transformed into *Escherichia coli* CC118 λ*pir*. The correct plasmids were then transformed into *E. coli* SM10 λ*pir* and then conjugated into EIB202. The transconjugants with the plasmids integrated into the chromosome by homologous recombination were selected on tryptic soy agar (TSA) medium containing kanamycin (Km, 50 μg/ml) or colistin (Col, 12.5 μg/ml). To complete the allelic exchange for in-frame deletions, double-crossover events were counter-selected on TSA plates containing 10% sucrose. All of the mutants were confirmed by PCR amplification of the respective DNA loci, and subsequent DNA sequencing of each PCR product. To construct pCX340-candidate effector, the DNA sequences of candidate effector genes were amplified with primers pCX340-candidate effector-F and pCX340-candidate effectors-R and ligated into the NdeI and KpnI restriction sites of pCX340 using One Step Cloning Kit (Vazyme). To construct pUTt-pBAD-effector-HA, the DNA sequence of candidate effectors genes fused with a C terminal HA tag were amplified with primers pUTt-candidate effector-F and pUTt-candidate effectors-R and the pBAD sequence was amplified with primers pBAD-F and pBAD-R. Two PCR fragments were ligated into the EcoRI and BamHI restriction sites of pUTt using ClonExpress MultiS One Step Cloning Kit (Vazyme). The plasmids were then electroporated into wild-type, ΔT3SS or ΔT6SS strains to generate strains expressing candidate effectors fusion with TEM or HA tag. The primers used in this study are listed in Table S3.

### Secretion assay

To examine the secretion of candidate effector proteins, overnight cultures of wild-type *E. piscicida* strain EIB202, the T3SS mutant, and the T6SS mutant expressing HA-tagged effectors were subcultured at 1:100 in DMEM and grown without shaking for 12 h at 30°C. L-Arabinose was added to induce the expression of effector-HA when the optical density (OD)_600_ was 0.6. The supernatant and the total bacterial proteins were prepared as described previously (Hou et al., 2017). The supernatant and the total bacterial pellet fraction were then separated by sodium dodecyl sulfate-polyacrylamide gel electrophoresis (SDS-PAGE) and transferred onto a polyvinylidene difluoride (PVDF) membrane for immunoblotting. Membranes were probed with mouse anti-HA antibody (1:2,000, Beyotime, China) and rabbit anti-RNAP antibody at 1:2,000 followed by horseradish per-oxidase (HRP)-conjugated goat anti-rabbit IgG at 1:5,000 (Beyotime, China).

### OMVs isolation

OMVs were purified from EIB202 as described previously with modifications (Chutkan et al., 2013). Briefly, the bacterial strains were grown in 100 ml of DMEM till OD_600_ of ~1.5 and the bacteria-free supernatant was collected by centrifugation at 5,000 × g for 10 min at 4°C. This supernatant was further filtered through a 0.45 μm filter and concentrated using a Vivaspin concentrator (GE Healthcare, molecular weight cutoff = 30 kDa). Subsequently, OMVs were pelleted by ultracentrifugation at 284,000 × g for 1.5 h at 4°C in a Beckman NVTTM65 rotor. Isolated OMVs were resuspended in PBS, transferred to the bottom of a 13 ml ultracentrifugation tube (Beckman Coulter) and adjusted to 45% OptiPrep (Sigma-Aldrich) in a final volume of 2 ml. Different OptiPrep/PBS layers were sequentially added as follows: 2 ml of 40%, 2 ml of 35%, 2 ml of 30%, 2 ml of 25%, and 1 ml of 20%. Gradients were centrifuged (284,000 × g, 16 h, 4°C) in a Beckman NVTTM65 rotor and fractions of equal volumes (1 ml) were removed sequentially from the top. Aliquots (9 μl) of the fractions were separated using SDS-PAGE in 12% gels, and analyzed using immunoblotting with anti-OmpA antibody (Figure S2A).Vesicle-containing fractions were selected and added to a centrifuge bottle with PBS, which were centrifuged at 284,000 × g for 1.5 h at 4°C. After removing the OptiPrep, OMVs were resuspended in 100 μl sterile without phenol red DMEM. Meanwhile, the OMV-free supernatants were further concentrated to 100 μl using a Vivaspin 20 concentrator (GE Healthcare). OMVs and OMV-free supernatants were separated by SDS-PAGE and immunoblotted with antibodies against OmpA, HA (CST, 2367) or RNAP (sc-47701).

### TEM1 protein translocation assay

As *E. piscicida* can infect and replicate in HeLa cells (Figure S1), the translocation of translational fusions between TEM1 and the *E. piscicida* candidate proteins was evaluated by detecting β-lactamase activity in infected HeLa cells as previously described (Xie et al., 2010). Briefly, TEM1 fusions of the effector candidates were transformed into wild-type *E. piscicida* by electroporation. Bacteria were grown in tryptic soy broth (TSB) overnight at 30°C, then diluted into DMEM and grown standing at 30°C until the OD_600_ reached 0.8. HeLa cells were then infected with strains harboring the TEM1 fusions at a MOI of 100. Infected cells were centrifuged at 600 × g for 10 min to initiate bacterial-cell contact, followed by incubation at 35°C for 3 h. Then, the cells were washed three times and incubated with fresh DMEM without serum for another 4 h. Cells were then washed three times with DMEM and loaded with the fluorescent substrate CCF2/AM (LiveBLAzer-FRET B/G loading kit; Invitrogen) in the β-lactamase loading solution supplemented with 15 mM Probenecid (Invitrogen). Cells were incubated in the dark for 120 min at 30°C and then observed under a Nikon A1R confocal microscope. Three independent random fields with at least 400 cells in each were chose and counted to determine the percentage of cells (TEM1-positive) emitting a blue fluorescence. The candidate genes whose positive percentage are more than 1% were selected for further identification.

### Fluorescence microscopy for the observation of effectors translocation

HeLa cells were seeded into wells of a 24-well plate (with coverslips at the bottom) at 2 × 10^5^ cells per well in DMEM for 18 h at 35°C in a 5% CO_2_ incubator. Then the HeLa cells were infected with wild-type EIB202 or the T6SS mutant harboring pUTt-pBAD-effector-HA as described above. Seven hours after infection, cells were washed with phosphate-buffered saline (PBS) and then fixed in 4% (wt/vol) paraformaldehyde for 10 min at room temperature. Fixed cells were washed in PBS, permeabilized and blocked with SSPBS (PBS containing 10% normal goat serum and 0.1% saponin) for 10 min at 37°C. After permeabilization and blocking, the cells were stained with anti-HA antibody (CST, 2367) overnight at 4°C, then washed with SPBS (PBS containing 0.05% saponin) three time for 5 min each. Then, the cells were incubated with the secondary antibody (Invitrogen, A11001) for 45 min at room temperature in the dark. Cell nuclei and bacteria were stained by DAPI for 15 min at 30°C. Fixed samples were observed under a confocal microscope (Nikon, A1R). In the OMV inhibition assay, cells were pretreated with the OMV internalization inhibitor, dynasore for 30 min before infection (80 μM), and the inhibitor was maintained throughout the course of infection.

### Virulence assessment in zebrafish

Three-month old healthy zebrafish (approximately 0.4 g, 4 cm) were purchased from a commercial fish farm (Shanghai, China) and reared in an aquarium for about 1 week. EIB202 and effector mutants were cultured in TSB medium overnight at 30°C and then diluted into fresh TSB and grown without shaking at 30°C to an OD_600_ of 0.8. The cells were then washed with PBS and suspended in PBS at 1.8 × 10^4^ CFU/ml. Then zebrafish were randomly divided into 11 groups (*n* = 35) and infected via intramuscular injection with 10 μl bacterial sample or PBS as a control. Fish mortality was recorded in each infection group was recorded over a period of 4 days. All animal experiments were approved by the Institutional Animal Care and Use Committee of East China University of Science and Technology.

### Statistical analysis

All experiments were performed three times (as indicated in the figure legends). Except for the mortality assay, in which a log-rank test was used to compare the survival distributions of the fish, all other statistical analyses were performed using The Student's *t*-test in the SPSS software (Version 11.5, SPSS Inc.). In all cases, the significance level was defined as ^*^p ≤ 0.01, ^**^p ≤ 0.05, or ^***^p ≤ 0.001.

## Results

### Identification of *E. piscicida* effector candidates based on macrophage infection-responsive genes

**Figure 1:**
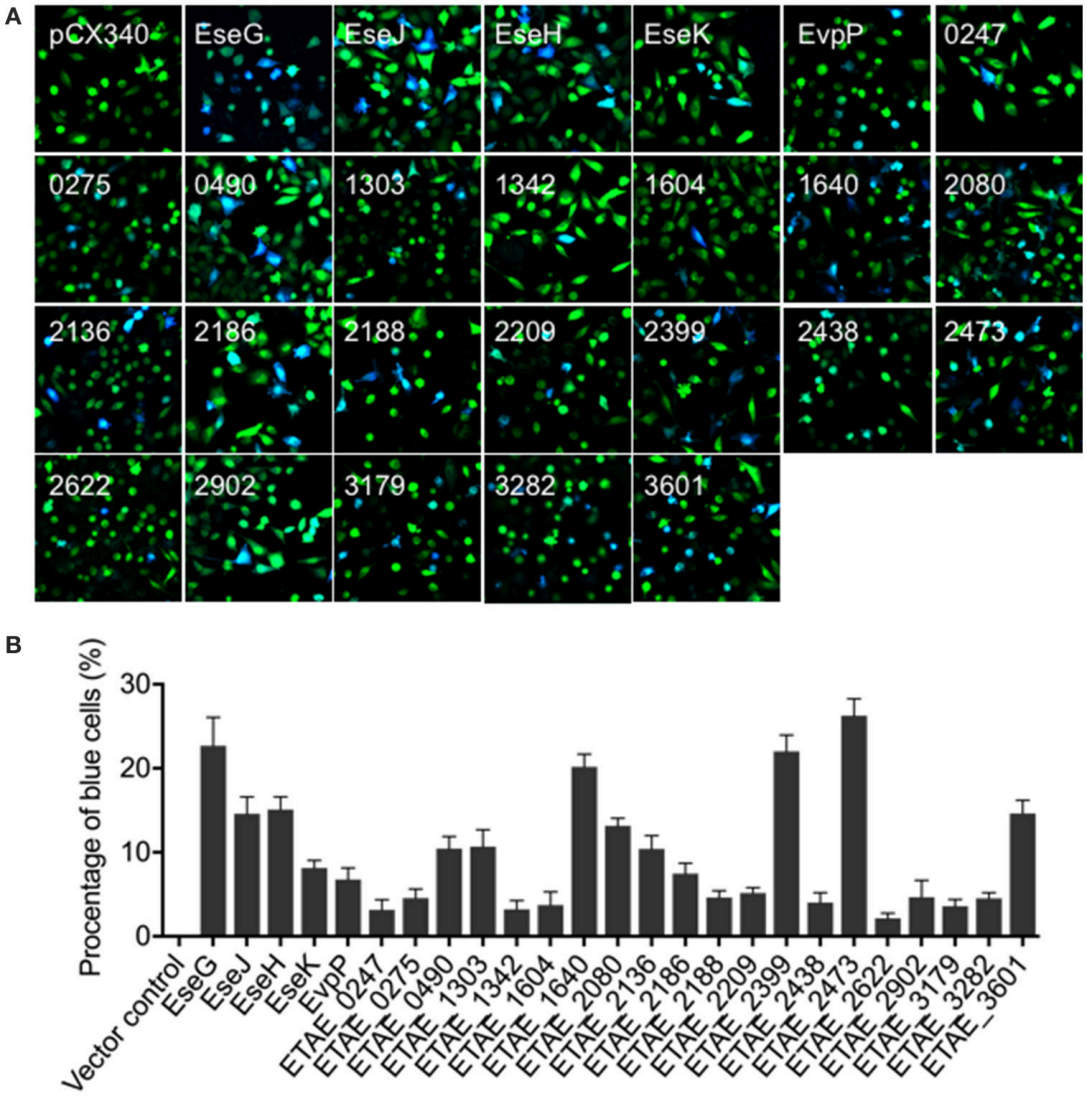
Translocation of *Edwardsiella piscicida* candidate effector proteins into HeLa cells**. (A)** HeLa cells were infected with wild-type *E. piscicida* carrying constructs expressing fusion proteins of β-lactamase TEM-1 with *E. piscicida* proteins at an MOI of 100 for 3 h, then incubated for an additional 4 h in fresh media. The infected cells were loaded with CCF4/AM, and the translocation of effectors was analyzed by fluorescence microscopy. Blue fluorescence indicates the positive translocation of effectors. Data are representative of three experiments, and representative microscopic images are shown. **(B)** The percentage of translocation- positive cells was calculated from three different fields, with at least 400 cells per fields in each condition. The graph shows the mean ± *SD* of three fields.

The overall objective of this study was to systematically identify novel *E. piscicida* effectors encoded inside and outside of the T3SS/T6SS gene cluster. To achieve this, we adapted a method based on a macrophage infection-responsive gene identification. We previously showed that, *E. piscicida* can infect macrophages and induce a comprehensive infection-responsive transcriptional profile (Zhang et al., 2016). We also showed that most T3SS and T6SS cluster genes were significantly up-regulated during the infection, and that these mainly (but not exclusively) enhanced bacterial pathogenesis *in vivo* (Zhang et al., 2016). To determine whether some of these infection-responsive genes encode potential *E. piscicida* effectors, 200 genes showing significantly transcriptionally-changed genes (including up-regulation and down-regulation) were selected based on RNA-seq data (Table S1) and tested for intracellular translocation using the TEM1 β-lactamase protein translocation reporter assay. As shown in Figure 1A, 25 out of 200 candidates were intracellularly translocated into HeLa cells, and are very likely *E. piscicida* effectors. Simultaneously, significant differences in translocation efficiency between the effectors were detected (Figure 1B). As expected, the five currently known *E. piscicida* effectors, namely EseG, EseJ, EseH, EseK, and EvpP, were included in this effector candidate database and showed positive intracellular translocation. These data indicated that the infection-based screening strategy used here can be used to narrow the screening range and effectively identify the candidate effectors.

### Secretion analysis of putative *E. piscicida* effector candidates

**Figure 2:**
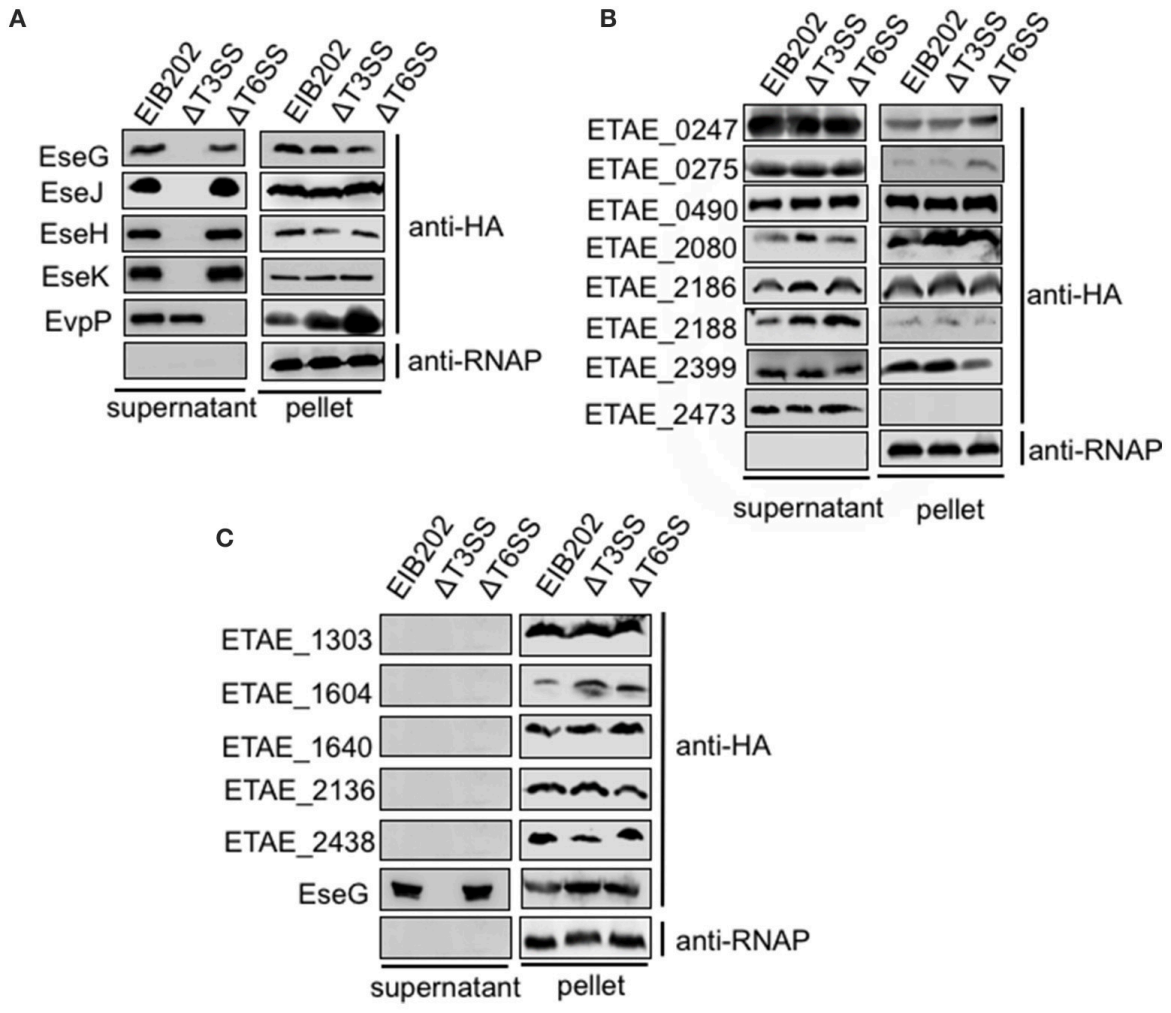
Secretion analysis of *Edwardsiella piscicida* candidate effector proteins. C-terminal HA-tagged effectors were expressed in the *E. piscicida* wild-type, ΔT3, and ΔT6SS strains and assayed for secretion. Some effectors were secreted in a T3 or T6-dependent manner **(A)**, some exhibited T3/T6SS-independent secretion **(B)**, and some were not secreted **(C)**. Proteins from bacterial pellets and secreted proteins in DMEM were separated by SDS-PAGE and blotted onto PVDF membranes for immunoblotting.

To determine whether the 25 translocation-positive candidates were substrates of *E. piscicida* T3SS or T6SS, we first test if their secretion are T3SS or T6SS-dependent. we generated constructs expressing the 25 effector genes with HA epitopes fused at the C termini. These constructs were introduced into wild-type *E. piscicida*, the T3SS mutant, or the T6SS mutant, and assayed by western blot for the presence of all the HA tagged proteins in the supernatants. Because seven of the 25 putative effectors were not expressed successfully in *E. piscicida* strains (data not shown), only the remaining 18 were subjected to secretion assays. Known T3SS effectors, EseG, EseJ, EseH and EseK were secreted by the wild type and T6SS mutant, not by T3SS mutant (Figure 2A), indicating that their secretion is T3SS-dependent. The secretion of EvpP, a known T6SS effector, was also confirmed to be secreted dependent on T6SS (Figure 2A). Another eight candidate effectors, including ETAE_0247, ETAE_0275, ETAE_0490, ETAE_2080, ETAE_2186, ETAE_2188, ETAE_2399, and ETAE_2473, showed similar levels of secretion from the wild-type, T3SS mutant and T6SS mutant, indicating that their secretion is not dependent on either T3SS or T6SS (Figure 2B). Finally, the remaining five proteins including ETAE_1303, ETAE_1604, ETAE_1640, ETAE_2136, and ETAE_2438 were not secreted at detectable levels by all of the strains, suggesting that secretion may not be necessary for these effector's intracellular translocation of these effectors (Figure 2C). Collectively, our data showed different secretion patterns for the identified translocation-positive candidate effectors, suggesting that these candidate effectors may be transported into cells via divergent secretion pathways that are not limited to T3SS and T6SS systems or may be in a secretion-independent manner.

### Confirmation of EseL and EseM as new T6SS-dependent candidate effectors

**Figure 3:**
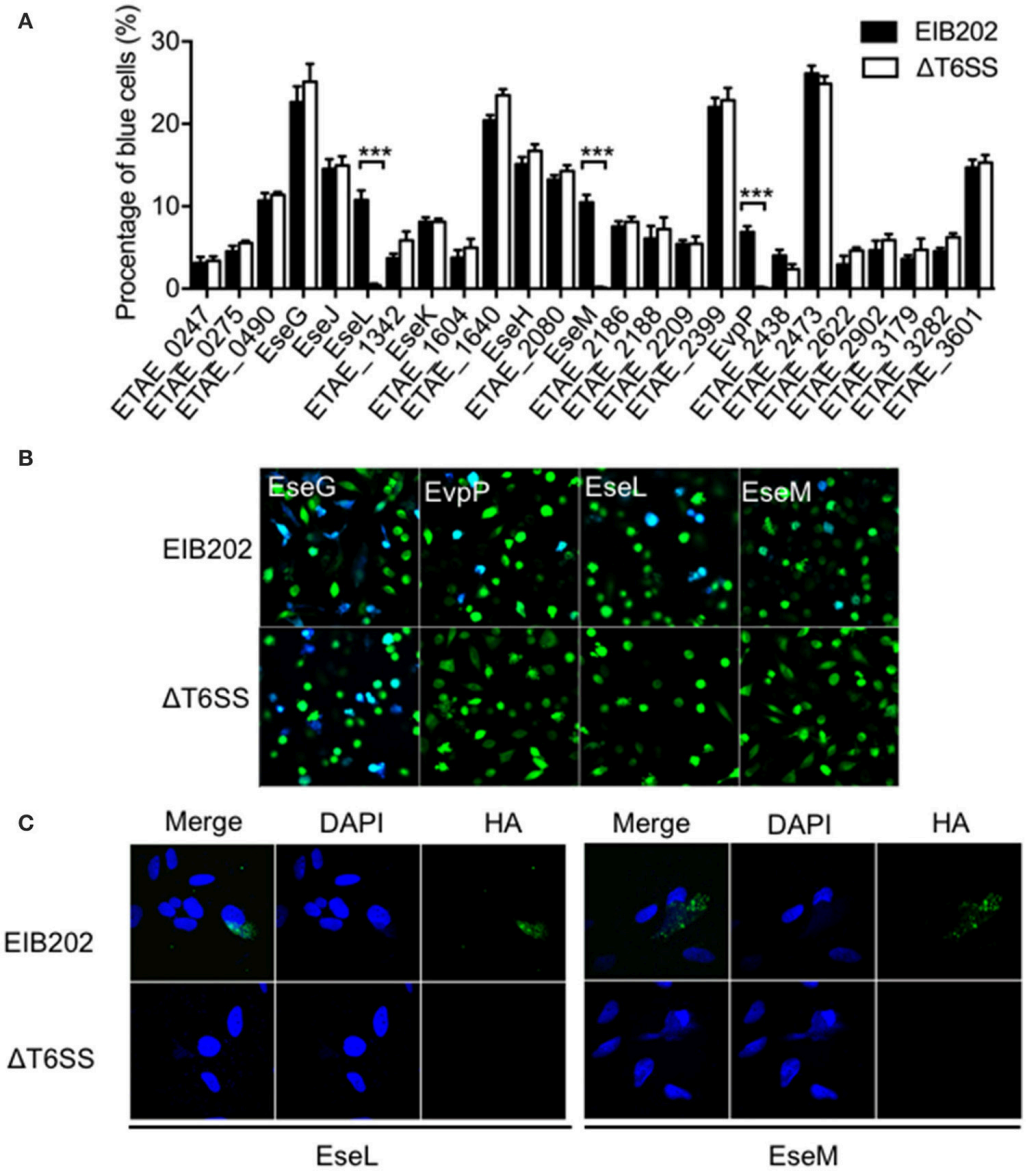
T6SS-dependent translocation of *Edwardsiella piscicida* candidate effectors into HeLa cells. **(A,B)** Wild-type *E. piscicida* EIB202 or the ΔT6SS mutant expressing fusion proteins of β-lactamase TEM-1 with *E. piscicida* proteins was used to infect HeLa cells. The infected cells were loaded with CCF4/AM, the percentage of translocation-positive cells was determined **(A)**, and three T6SS-dependent effector translocations are shown in representative fluorescence micrographs **(B)**. **(C)** Wild-type *E. piscicida* EIB202 or the ΔT6SS mutant carrying constructs expressing HA-tagged proteins were used to infect HeLa cells at an MOI of 100 for 3 h and then incubated for an additional 4 h in fresh media. Cells were fixed, permeabilized, and immunostained with DAPI (middle) and HA (right). An overlay of the two images is shown on the left.

To find putative T6SS-dependent effectors among the newly identified translocation-positive proteins, we used the TEM1 fusion system to compare the translocation of 24 effector candidates from the wild type and T6SS mutant. As shown in Figures 3A,B, in addition to the known T6SS effector EvpP, two other candidate effectors, ETAE_1303 (named as EseL) and ETAE_2136 (named as EseM) also showed a significantly reduced intracellular transport effectiveness in the T6SS mutant relative to that in the wild type. Consistently, the immunofluorescence assay showed HA-tagged EseL or EseM in the cytoplasm of cells infected by the wild type but not by T6SS mutant (Figure 3C), confirming that the translocation of the two candidate effectors is T6SS-dependent. Collectively, we identified three T6SS effectors from the macrophage infection-responsive *E. piscicida* genes, including one known effector and two new ones.

### Confirmation of EseN, EseO, and EseP as new OMV-dependent candidate effectors

**Figure 4:**
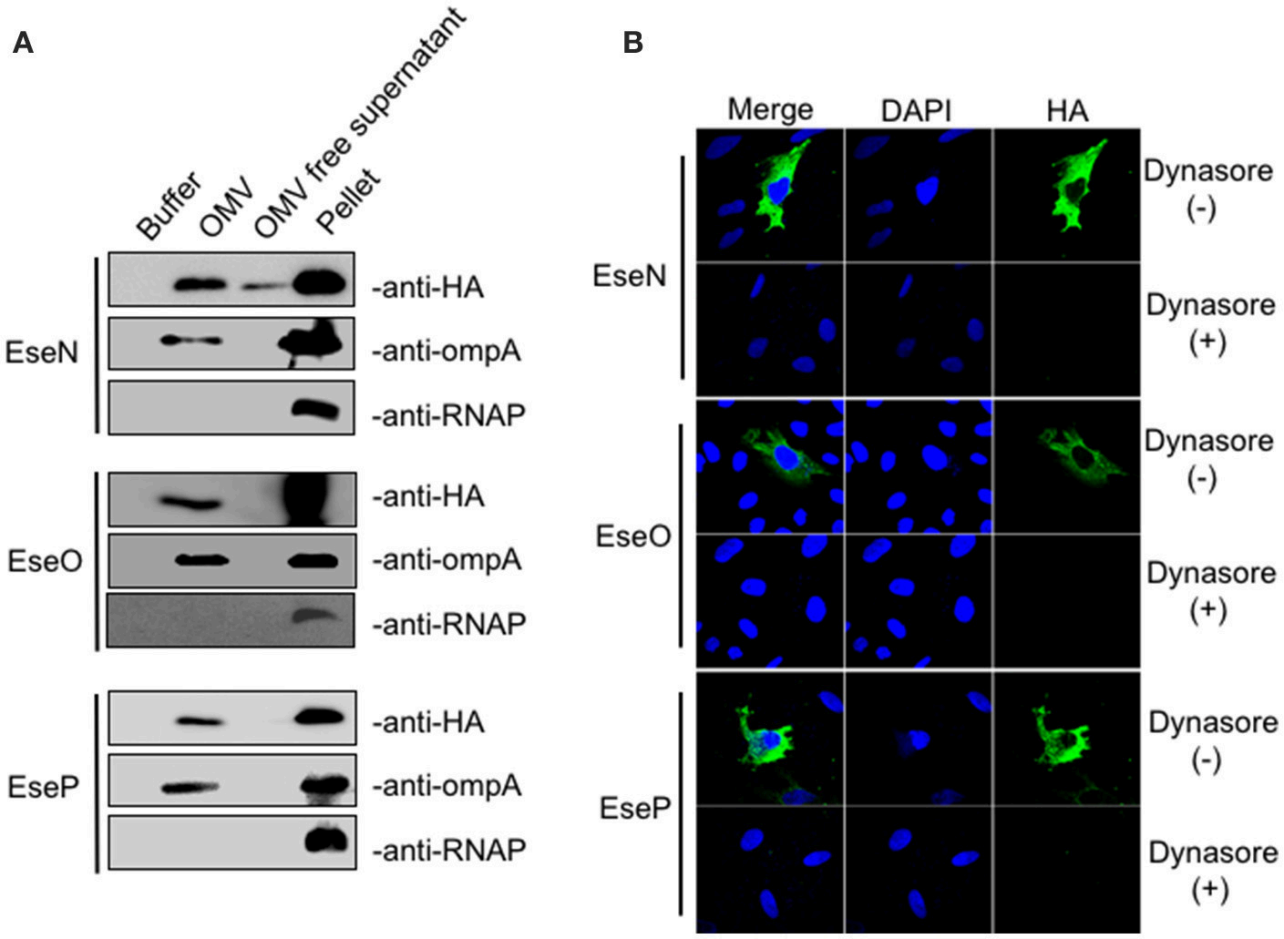
Secretion and translocation analysis of outer membrane vesicles (OMV)-dependent *Edwardsiella piscicida* candidate effectors**. (A)** *E. piscicida* expressing HA-tagged EseN, EseO, or EseP were cultivated in DMEM medium for 12 h. OMV fractions and bacterial cell pellets were prepared as described in section Materials and Methods and immunoblotted using anti-HA, anti-OmpA and anti-RNAP antibody. OmpA, a highly expressed outer membrane protein, was used as a control for the presence of OMVs. RNAP, a cytosolic protein, was used to verify that no bacterial lysis occurred during the OMV isolation procedure. **(B)** HeLa cells were pretreated with dynasore (80 μM) or DMSO as a control for 30 min, and the treatments were maintained throughout the course of infection. Cells were then infected with *E. piscicida* strains expressing HA-tagged EseN, EseO, or EseP for 7 h. Cells were fixed, permeabilized, and immunostained with DAPI, HA, and OmpA.

In addition to T3SS and T6SS systems, OMVs have been identified as another means to deliver bacteria virulence factors into host cells (Yoon et al., 2011). Considering that eight of the 25 candidate effectors we discovered in this work were not dependent on either T3SS or T6SS for secretion, we further examined the possibility of their secretion and translocation through OMVs. OMV fractions were extracted from the culture supernatants of *E. piscicida* strains expressing HA-tagged effectors by ultracentrifugation and analyzed by TEM (transmission electron microscope; Figure S4). The presence of effectors in the OMVs fraction was then analyzed by western blot. The outer membrane protein OmpA was used to indicate whether the fraction contained OMVs. As shown in Figure 4A, ETAE_0247 (named EseN), ETAE_0490 (named EseO), and ETAE_2080 (named EseP) were prominent in the OMV fraction, suggesting that the three effectors may be secreted mainly through OMVs. Furthermore, intracellular transport of EseN, EseO, and EseP was investigated after pretreatment with dynasore, an OMVs internalization inhibitor or without pretreatment (Figure S5). Immunofluorescence assay showed that HA-tagged EseN, EseO, and EseP can be transported into the cells in the absence of dynasore, whereas the addition of dynasore significantly inhibits this process (Figure 4B), indicating that the intracellular translocation of EseN, EseO, and EseP is dependent on OMVs. Thus, based on the fact that OMVs are critical for both the secretion and intracellular transport of the three proteins, EseN, EseO, and EseP were identified as three previously undiscovered OMVs-dependent candidate effectors of *E. piscicida*.

### Functional analysis of the identified candidate effectors

**Figure 5:**
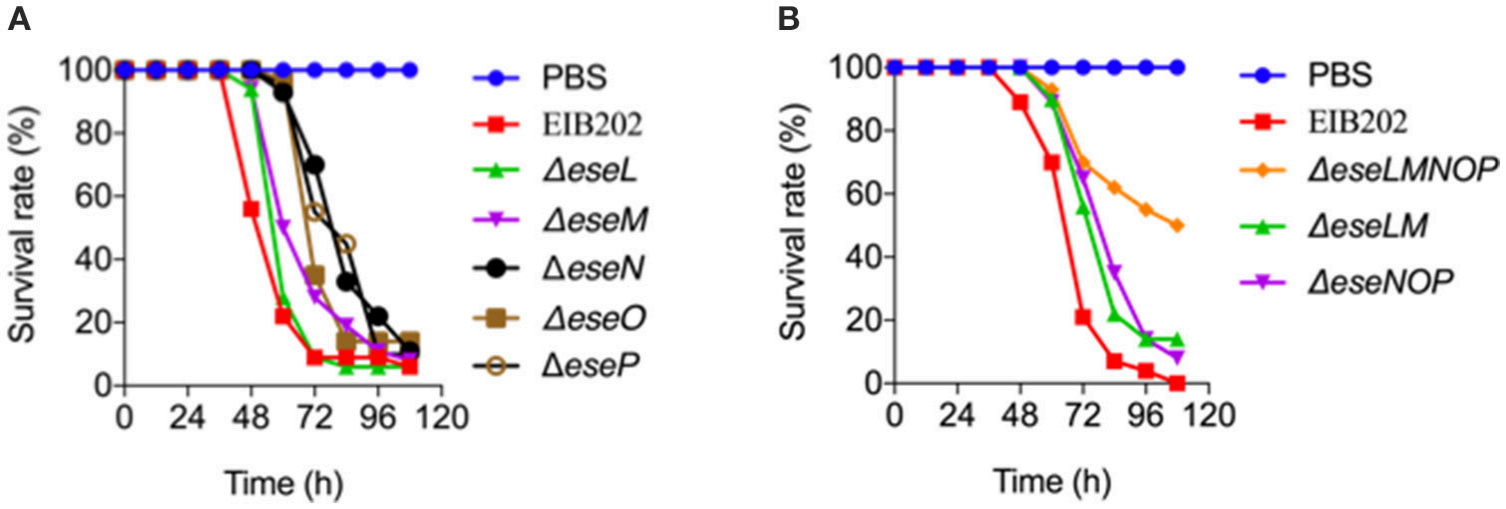
Survival of zebrafish infected with the indicated candidate effector mutants of *Edwardsiella piscicida*. Zebrafish were intramuscularly injected with wild-type *E. piscicida* EIB202, Δ*eseL*, Δ*eseM*, Δ*eseN*, Δ*eseO*, Δ*eseP* **(A)**, or Δ*eseLM*, Δ*eseNOP*, Δ*eseLMNOP* **(B)** at 180 CFU per fish. PBS was used as a control. The survival of zebrafish was determined 4 days after injection, *n* = 35 fish per group.

Here, 10 *E. piscicida* candidate effectors were identified from a collection of bacterial genes that responded significantly to macrophage infection. Of these, five were known effectors and five were new ones. Known *E. piscicida* effectors have been shown to participate in regulating intracellular infection processes. For example, EseG is involved in microtubule depolymerization (Xie et al., 2010; Fang et al., 2016), EseJ helps *E. piscicida* proliferate in the cell (Xie et al., 2015), EseH inhibits MAPK activation via phosphothreonase activity to promote infection (Hou et al., 2017), and EvpP promotes bacterial colonization by inhibiting NLRP3 inflammasome activation (Chen et al., 2017). To gain insight into the function of the five new *E. piscicida* candidate effectors identified in this work, a bioinformatics analysis was conducted. The results indicated that the two T6SS candidate effectors, EseL and EseM encode a class of conserved major cold shock proteins, whereas the other three OMV-dependent candidate effectors, EseN, EseO, and EseP, were annotated as copper-zinc superoxide dismutase, putative cytochrome and lipoprotein, respectively (Table 1). Copper-zinc superoxide dismutase in Mycobacterium tuberculosis was reported to protect bacteria from oxidative burst of phagocytes (Piddington et al., 2001) and lipoprotein of Gram-positive bacteria were found to play important roles in the immune response and contribute to their virulence (Nguyen and Götz, 2016). To further investigate the roles of the five new effectors in *E. piscicida* virulence, we generated five single gene-deletion mutants, Δ*eseL*, Δ*eseM*, Δ*eseN*, Δ*eseO*, and Δ*eseP* and three multiple gene-deletion mutants, Δ*eseLM*, Δ*eseNOP*, and Δ*eseLMNOP*, of *E. piscicida*. Mutations in these genes did not affect strain growth (Figure S2) or activity of the T3SS, T6SS, or OMV secretion pathways (Figure S3). Zebrafish were infected with the wild type, Δ*eseL*, Δ*eseM*, Δ*eseN*, Δ*eseO*, Δ*eseP*, Δ*eseLM*, Δ*eseNOP*, and Δ*eseLMNOP* by intraperitoneal injection at 180 colony-forming units (CFU) per fish, respectively, and the survival of fish was recorded at different time intervals after infection. As shown in Figure 5A, although all the single gene-deletion mutants didn't show significant attenuation in zebrafish when compared with the wild type, Δ*eseM*, Δ*eseN*, and Δ*eseO* showed diminished virulence in the first 84 h. Two multiple Δ*eseLM* and Δ*eseNOP* also showed increased survival comparing to wild type (Figure 5B). In contrast, significant attenuation in zebrafish was observed for Δ*eseLMNOP* when compared to that of the wild-type strain (Figure 5B, *P* < 0.001). Taken together, these data indicated that although the role of a single effector in the process of *E. piscicida* infection is not obvious, the coordinated action of multiple effectors may contribute to full bacterial virulence.

**Table 1:** Schematic representation of secreted effector proteins (Ese proteins) of *Edwardsiella piscicida* that indicates the corresponding locus, predicted functions, and translocation pathways of these effectors.

**Locus**	**Gene**	**Annotation**	**Secretion/Translocation pathway**	**References**
ETAE_0866	*eseG*	Two-component sensor/regulator	T3SS	Xie et al., 2010
ETAE_0888	*eseJ*	Putative TTSS effector protein	T3SS	Xie et al., 2015
ETAE_1757	*eseH*	Hypothetical protein	T3SS	Hou et al., 2017
ETAE_1586	*eseK*	Hypothetical protein	T3SS	Cao et al., 2017
ETAE_1303	*eseL*	Major cold shock protein	T6SS	This work
ETAE_2136	*eseM*	Major cold shock protein	T6SS	This work
ETAE_2428	*evpP*	Type VI secretion system protein	T6SS	Chen et al., 2017
ETAE_0247	*eseN*	Copper-zinc superoxide dismutase	OMV	This work
ETAE_0490	*eseO*	Putative cytochrome	OMV	This work
ETAE_2080	*eseP*	Lipoprotein	OMV	This work

## Discussion

Many bacterial pathogens can survive and multiply within a variety of eukaryotic cells, which is believed to be important for their infection (Hybiske and Stephens, 2008). Once inside a cell, the bacterium needs to promptly adapt to the unique host environment (Flannagan et al., 2009). Pathogens have evolved precise regulation strategies to remodel an infection-responsible gene expression profile upon infection through the delicate interplay of virulence factors (Poole, 2012). For example, *Salmonella enterica* expresses a characteristic intracellular transcriptomic signature during infection that involves the up-regulation of *Salmonella* pathogenicity island 1 and 2 (Cirillo et al., 1998; Hautefort et al., 2008), and *Burkholderia pseudomallei* up-regulates a T6SS locus upon invasion of macrophages (Shalom et al., 2007). In this work, based on the assumption that bacterial effectors might be transcriptionally regulated during the bacterial infection cycle, we implemented an infection transcriptome-based screening method that reduced the screening pool from genome-encoded more than 3,000 genes to 200 genes that responded significantly to infection and identified a total of 25 effector candidates from the reduced gene collection, with a positive ratio of more than 10%. Because virulence gene expression in response to intracellular infection signals is very common in pathogens, the strategy used in this work has the potential to be readily applied in identifying critical effectors in other bacterial pathogens.

Bacterial pathogens have evolved diverse secretory machineries to secrete virulence factors from the cytosol of the bacteria into host cells or the host environment. In general, bacterial protein secretion apparatuses can be divided into nine classes (Type 1–9 secretion system), based on their structures, functions, and specificity (Abby et al., 2016). Some systems are conserved in all classes of bacteria and secrete a broad array of substrates, while others are only found in a small number of bacterial species and/or are specific to only one or a few proteins. In *E. piscicida*, only type III and type VI secretion system have been identified and well-studied (Zheng and Leung, 2007; Yang et al., 2012). And a potential type IV secretion system encoded on the plasmid of *E. piscicida* was recently identified to play an important role in pEIB202 horizontal transfer (Liu et al., 2017). More importantly, systematic identification of bacterial effectors using different secretion and transport pathways has been very challenging. In this work, our screening for candidate effectors was based on a real pathogen-host interaction model, which increased the sensitivity and effectiveness of effector screening. Using this approach, we identified 25 candidate effectors, including all five known effectors, greatly expanding the documented effectors repertoire of *E. piscicida*. The candidate effectors identified using this method were from different classes, including T3SS, T6SS, or OMV-dependent effectors, suggesting that *E. piscicida* requires the collaboration of effectors that use divergent pathways to promote infection. besides, some candidate effectors identified in our work are secreted independent of either T3SS or T6SS (Figure 2B), suggesting that other secretion systems like T4SS or unknown secretion mechanism may be involved in effector's translocation in *E. piscicida*.

Bacteria have also evolved sophisticated signal transduction systems to sense and adapt to their environment. The field of microbial pathogenesis offers many examples of coordinate expression of virulence determinants controlled at the transcriptional level in response to physico-chemical changes in the various habitats encountered by the pathogen (Mekalanos, 1992). There is also accumulating evidence that some virulence traits may be induced by more specific signals resulting from host-pathogen cross-talk, acid environment or oxidant stress. For example, contact between *Yersinia pseudotuberculosis* and mammalian cells induces expression of YopE and the subsequent polarized transfer of the YopE cytotoxin into the cytosol of the eukaryotic cell (Rosqvist et al., 1994). Similarly, studies with *Salmonella* have shown that bacterial effector was translocated into cytosol by sensing acid pH (Yu et al., 2010). In this work, five of 25 effectors are not secreted into the supernatants at detectable level (Figure 2C), indicating that some unique infectious signals might be required to induce effectors' secretion.

Up to 100 different effector proteins may be delivered into individual host cells by a single bacterium (Kenny and Valdivia, 2009). Multiple effectors, especially those using T3SS and T4SS systems, cooperate with each other to directly mimic, intercept, or modify the function of key host factors engaged in a wide range of cellular processes, including innate immune signaling, cytoskeletal dynamics, membrane trafficking, phosphoinositide lipid metabolism, and cell signaling (Dean, 2011). Unlike the T3SS and T4SS systems, relatively few effectors have been reported to use T6SS or OMV-mediated secretion pathways, partly because of the lack of useful effector-screening strategies. In this work, we verified two new T6SS candidate effectors, EseL and EseM, and three OMV candidate effectors, EseN, EseO, and EseP. Further analysis of their roles in bacterial virulence showed that the four candidate effectors collectively contributed to the pathogen's virulence, and that any mutation in an individual effector gene had a minor effect. This result suggests that each putative effector may be part of a comprehensive process and thus have limited individual effect during infection. It also further highlights the importance of knowing the full effector repertoire of pathogens.

## Author contributions

LZ: performed the majority of the experiments; ZJ: undertook OMV effector-related experiments; SF and YH: participated in T3SS and T6SS effector-related experiments; QL and LZ: conceived the study, analyzed all the data, and wrote the manuscript; YZ, DY, and QW: critically revised the manuscript; All the authors discussed the results and commented on the manuscript.

### Conflict of interest statement

The authors declare that the research was conducted in the absence of any commercial or financial relationships that could be construed as a potential conflict of interest.
