# Supplementary material for: Systematic Identification of Intracellular-Translocated Candidate Effectors in Edwardsiella piscicida
Source: Front Cell Infect Microbiol. 2018 Feb 16;8:37. doi: 10.3389/fcimb.2018.00037 (PMC5820615; doi:10.3389/fcimb.2018.00037)
Supplement: Supplementary file 1 [file Presentation1.PDF]

TABLE S1 The 200 gene candidates for TEM1 protein translocation assay

gene	Function	Percentage of blue cells (%)	FRET result
ETAE_0047	conserved hypothetical protein	0	negative
ETAE_0084	2-amino-3-ketobutyrate coenzyme A ligase	0	negative
ETAE_0085	L-threonine 3-dehydrogenase	0	negative
ETAE_0132	conserved hypothetical protein	0	negative
ETAE_0134	gluconate transporter, low affinity GNT 1 system	0	negative
ETAE_0135	gluconate kinase 1	0	negative
ETAE_0137	zinc/cadmium/mercury/lead-transporting ATPase	0	negative
ETAE_0156	peptidase E	0	negative
ETAE_0176	DNA-directed RNA polymerase, beta subunit/160 kD subunit	0	negative
ETAE_0231	DNA damage response protein	0	negative
ETAE_0247	copper-zinc superoxide dismutase	3.2	positive
ETAE_0275	hypothetical protein	4.5	positive
ETAE_0292	hypothetical protein	0	negative
ETAE_0311	aspartate ammonia-lyase	0	negative
ETAE_0338	fumarate reductase flavoprotein subunit	0	negative
ETAE_0362	hypothetical protein	0	negative
ETAE_0372	cell morphogenesis/cell wall metabolism regulator	0	negative
ETAE_0411	ATP-dependent RNA helicase	0	negative
ETAE_0435	galacturonate transporter	0	negative
ETAE_0436	glucuronate isomerase	0	negative
ETAE_0438	altronate hydrolase	0	negative
ETAE_0472	3,4-dihydroxy-2-butanone 4-phosphate synthase	0	negative
ETAE_0474	conserved hypothetical protein	0	negative
ETAE_0490	putative cytochrome	10.5	positive
ETAE_0512	trypsin-like serine proteases(degQ)	0	negative
ETAE_0530	hypothetical protein	0	negative
ETAE_0555	conserved hypothetical protein	0	negative
ETAE_0578	Na+/H+ antiporter	0	negative
ETAE_0586	putative outer membrane protein	0	negative
ETAE_0666	conserved inner membran protein	0	negative
ETAE_0696	outer membrane protein S1 precuror	0	negative
ETAE_0729	conserved hypothetical protein	0	negative
ETAE_0732	hypothetical protein	0	negative
ETAE_0770	putative alcohol dehydrogenase	0	negative
ETAE_0851	putative sulfite oxidase	0	negative
ETAE_0853	putative guanine aminohydrolase	0	negative
ETAE_0866	two-component sensor/regulator	22.7	positive
ETAE_0867	type III secretion system chaperone protein B(ESCB)	0	negative
ETAE_0871	type III secretion low calcium response	0	negative

	chaperone(escA)		
ETAE_0888	putative TTSS effector protein	14.6	positive
ETAE_0903	hypothetical protein	0	negative
ETAE_0904	lactose/L-arabinose transport system permease protein	0	negative
ETAE_0906	putative extracellular solute-binding protein	0	negative
ETAE_0913	putative formate acetyltransferase 2	0	negative
ETAE_0922	hypothetical protein	0	negative
ETAE_0941	hypothetical protein	0	negative
ETAE_0943	putative amino acid permease	0	negative
ETAE_1002	hypothetical protein	0	negative
ETAE_1029	hypothetical protein	0	negative
ETAE_1030	type 1 fimbrial protein	0	negative
ETAE_1031	fimbrial chaperon protein	0	negative
ETAE_1036	gluconate:H+ symporter, GntP family	0	negative
ETAE_1050	putative NADH oxidase	0	negative
ETAE_1057	hydroxyisourate hydrolase	0	negative
ETAE_1063	hypothetical protein	0	negative
ETAE_1142	manganese transport protein	0	negative
ETAE_1157	hypothetical protein	0	negative
ETAE_1158	hypothetical protein	0	negative
ETAE_1159	hypothetical protein	0	negative
ETAE_1163	anti holin-like protein（LrgB）	0	negative
ETAE_1164	anti holin-like protein（LrgA）	0	negative
ETAE_1206	sialic acid synthase	0	negative
ETAE_1217	hypothetical protein	0	negative
ETAE_1276	hypothetical protein	0	negative
ETAE_1282	hypothetical protein	0	negative
ETAE_1289	hypothetical protein	0	negative
ETAE_1303	major cold shock protein	10.7	positive
ETAE_1304	hypothetical protein	0	negative
ETAE_1311	putative integral membrane protein	0	negative
ETAE_1322	hypothetical protein	0	negative
ETAE_1324	peptidoglycan glycosyltransferase	0	negative
ETAE_1342	hypothetical protein	3.2	positive
ETAE_1412	hypothetical protein	0	negative
ETAE_1438	holliday junction resolvasome, endonuclease subunit	0	negative
ETAE_1473	hypothetical protein	0	negative
ETAE_1507	hypothetical protein	0	negative
ETAE_1528	outer membrane protein W	0	negative
ETAE_1544	hypothetical protein	0	negative
ETAE_1572	fructosamine kinase	0	negative
ETAE_1573	hypothetical protein	0	negative

ETAE_1574	lytic transglycosylase catalytic	0	negative
ETAE_1586	hypothetical protein	8.1	positive
ETAE_1604	hypothetical protein	3.7	positive
ETAE_1629	hypothetical protein	0	negative
ETAE_1638	hypothetical protein	0	negative
ETAE_1640	putative prophage protein	20.2	positive
ETAE_1708	fumarate hydratase	0	negative
ETAE_1714	dethiobiotin synthetase	0	negative
ETAE_1716	transcriptional antiterminator	0	negative
ETAE_1732	hypothetical protein	0	negative
ETAE_1733	outer membrane protein induced after carbon starvation	0	negative
ETAE_1743	hypothetical protein	0	negative
ETAE_1745	universal stress protein	0	negative
ETAE_1752	hypothetical protein	0	negative
ETAE_1757	hypothetical protein	15.1	positive
ETAE_1765	hypothetical protein	0	negative
ETAE_1766	putative pepetidase	0	negative
ETAE_1771	D-lactate dehydrogenase	0	negative
ETAE_1772	hypothetical protein	0	negative
ETAE_1780	hypothetical protein	0	negative
ETAE_1789	phosphoenolpyruvate synthase	0	negative
ETAE_1798	hemin transport protein	0	negative
ETAE_1799	hemin-binding periplasmic protein	0	negative
ETAE_1826	outer membrane protein	0	negative
ETAE_1854	hypothetical protein	0	negative
ETAE_1855	hypothetical protein	0	negative
ETAE_1893	site-specific recombinase	0	negative
ETAE_1897	periplasmic stress adaptor protein	0	negative
ETAE_1903	hypothetical protein	0	negative
ETAE_1931	hypothetical protein	0	negative
ETAE_1932	hypothetical protein	0	negative
ETAE_1938	hypothetical protein	0	negative
ETAE_1943	putative phage glucose translocase	0	negative
ETAE_1971	vibrioferrin biosynthesis protein	0	negative
ETAE_1974	ferric vibrioferrin receptor	0	negative
ETAE_1994	vitamin B12 biosynthetic protein	0	negative
ETAE_2010	sensory histidine kinase in two-component regulatory system with AtoC	0	negative
ETAE_2036	PEBP family protein	0	negative
ETAE_2037	kinesin family	0	negative
ETAE_2038	hypothetical protein	0	negative
ETAE_2069	hypothetical protein	0	negative

ETAE_2080	lipoprotein	20.2	positive
ETAE_2136	major cold shock protein	10.4	positive
ETAE_2139	putative integrase	0	negative
ETAE_2141	hypothetical protein	0	negative
ETAE_2167	3-deoxy-manno-octulosonate cytidylyltransferase	0	negative
ETAE_2170	cold-shock domain family protein	0	negative
ETAE_2185	hypothetical membran protein	0	negative
ETAE_2186	thioredoxin (H-type,TRX-H)	7.5	positive
ETAE_2187	putative cytochrome c-type biogenesis protein	0	negative
ETAE_2188	hypothetical protein	4.6	positive
ETAE_2209	cold shock protein	5.2	positive
ETAE_2228	putative outer membrane protein	0	negative
ETAE_2259	hypothetical protein	0	negative
ETAE_2263	probable serine transporter	0	negative
ETAE_2272	hypothetical protein	0	negative
ETAE_2326	transcriptional regulator, AraC family	0	negative
ETAE_2327	ATP-dependent RNA helicase	0	negative
ETAE_2396	hypothetical protein	0	negative
ETAE_2399	rhodanese-like protein	22	positive
ETAE_2405	hypothetical protein	0	negative
ETAE_2410	colicin V production protein	0	negative
ETAE_2419	acid shock protein precursor	0	negative
ETAE_2422	5-methylaminomethyl-2-thiouridine methyltransferase	0	negative
ETAE_2428	type VI secretion system protein EvpP	6.8	positive
ETAE_2429	type VI secretion system protein EvpA	0	negative
ETAE_2430	type VI secretion system protein EvpB	0	negative
ETAE_2431	type VI secretion system protein EvpC	0	negative
ETAE_2432	type VI secretion system protein EvpD	0	negative
ETAE_2433	type VI secretion system protein EvpE	0	negative
ETAE_2434	type VI secretion system protein EvpF	0	negative
ETAE_2436	type VI secretion system protein EvpH	0	negative
ETAE_2437	type VI secretion system protein EvpI	0	negative
ETAE_2438	type VI secretion system protein EvpJ	4	positive
ETAE_2439	type VI secretion system protein EvpK	0	negative
ETAE_2441	type VI secretion system protein EvpM	0	negative
ETAE_2473	phage-related protein, tail component	26.3	positive
ETAE_2476	phage tail component L-like protein	0	negative
ETAE_2477	phage-related minor tail protein	0	negative
ETAE_2493	hypothetical protein	0	negative
ETAE_2497	hypothetical protein	0	negative
ETAE_2509	carbon starvation protein, predicted membrane protein	0	negative

ETAE_2546	hypothetical protein	0	negative
ETAE_2622	hypothetical protein	2.2	positive
ETAE_2655	hypothetical protein	0	negative
ETAE_2658	putative crotonobetaine/carnitine-CoA ligase	0	negative
ETAE_2676	hypothetical protein	0	negative
ETAE_2678	hypothetical protein	0	negative
ETAE_2694	hypothetical protein	0	negative
ETAE_2707	hypothetical protein	0	negative
ETAE_2807	hypothetical protein	0	negative
ETAE_2902	electron transport protein	4.7	positive
ETAE_2909	formate hydrogenlyase regulatory protein HycA	0	negative
ETAE_2913	hypothetical protein	0	negative
ETAE_2942	hypothetical protein	0	negative
ETAE_2976	hypothetical protein	0	negative
ETAE_3001	hydrogenase-1 small subunit	0	negative
ETAE_3008	inner membrane protein	0	negative
ETAE_3017	hypothetical protein	0	negative
ETAE_3019	hypothetical protein	0	negative
ETAE_3041	hypothetical protein	0	negative
ETAE_3077	transposase, IS21 family	0	negative
ETAE_3112	hypothetical protein	0	negative
ETAE_3124	aspartate carbamoyltransferase regulatory subunit	0	negative
ETAE_3178	aspartate/ornithine carbamoyltransferase family protein	0	negative
ETAE_3179	hypothetical protein	3.6	positive
ETAE_3180	hypothetical protein	0	negative
ETAE_3282	hypothetical protein	4.5	positive
ETAE_3357	rhamnose transport system substrate-binding protein	0	negative
ETAE_3369	hypothetical protein	0	negative
ETAE_3372	hypothetical protein	0	negative
ETAE_3417	putative bacteriophage protein	0	negative
ETAE_3418	hypothetical protein	0	negative
ETAE_3452	periplasmic stress adaptor protein	0	negative
ETAE_3515	ATP-binding components	0	negative
ETAE_3516	high-affinity D-ribose transport protein	0	negative
ETAE_3547	hypothetical protein	0	negative
ETAE_3552	RNase P protein component	0	negative
ETAE_3559	hypothetical protein	0	negative
ETAE_3601	hypothetical protein	14.6	positive
ETAE_3634	hypothetical protein	0	negative

TABLE S2 Strains and plasmids used in this study

Strains and plasmids	Description	Reference
Edwardsiella piscicida		
EIB202	*Edwardsiella piscicida* wild-type strain, Col^r, Cm^r	CCTCC No: M208068
ΔT3SS EIB202	EIB202, deletion of *eseB-eseD*	Yang et al., 2015
ΔT6SS EIB202	EIB202, delection of *evpA-evpB*	Yang et al., 2015
Δ*eseL*	EIB202, in-frame deletion of ETAE_1303	This study
Δ*eseM*	EIB202, in-frame deletion of ETAE_2136	This study
Δ*eseN*	EIB202, in-frame deletion of ETAE_0247	This study
Δ*eseO*	EIB202, in-frame deletion of ETAE_0490	This study
Δ*eseP*	EIB202, in-frame deletion of ETAE_2080	This study
Δ*eseLM*	EIB202, in-frame deletion of ETAE_1303 and ETAE_2136	This study
Δ*eseNOP*	EIB202, in-frame deletion of ETAE_0247, ETAE_0490 and ETAE_2080	This study
Δ*eseLMNOP*	EIB202, in-frame deletion of ETAE_1303, ETAE_2136, ETAE_0247, ETAE_0490 and ETAE_2080	This study
Escherichia coli		
DH5α	Δ(*lacZYA-argF*) U169 (Φ80 *LacZ ΔM15*)	
cc118 *λpir*	*λpir* lysogen Δ(*ara-leu*) *araD* Δ(*lacX74*) *phoA20 thi-1 rpoB argE* (*am*)*recA1*	
SM10 *λpir*	*thi thr leu tonA lacy supE recA*::*RP4-2-Tc*::*Mu λpir Kan*^{r}	
**Plasmids**		
pMD19-T	PCR cloning vector, Amp^r	TaKaRa
pDM4	Suicide plasmid, pir dependent, R6K, SacBR, Cm^r	
pDMK	pDM4 derivative with Km resistance, Km^r	
pCX340	pBR322 derivative, cloning vector used to fuse EIB202 effectors to the mature form of TEM-1 β-lactamase, Tet^r	Xie et al., 2010
pCX340-*eseG*	pCX340 derivative containing *eseG*	Xie et al., 2010
pCX340-*eseJ*	pCX340 derivative containing *eseJ*	Xie et al., 2015
pCX340-*eseH*	pCX340 derivative containing *eseH*	Hou et al., 2017
pCX340-*eseK*	pCX340 derivative containing *eseK*	Cao et al., 2017
pCX340-*evpP*	pCX340 derivative containing *evpP*	Chen et al., 2017
pCX340-candidate genes	pCX340 derivative containing 200 candidate genes	This study
pUTt	Ori from pBR322, Amp^r	Yang et al., 2015
pUTt-pBAD-*eseL*-HA	pUTt derivative containing fragment of L-ara promoter and *eseL*-HA fusion, Amp^r	This study
pUTt-pBAD-*eseM*-HA	pUTt derivative containing fragment of L-ara promoter and *eseM*-HA fusion, Amp^r	This study

pUTt-pBAD-*eseN*-HA	pUTt derivative containing fragment of L-ara promoter and *eseN*-HA fusion, Amp^r	This study
pUTt-pBAD-*eseO*-HA	pUTt derivative containing fragment of L-ara promoter and *eseO*-HA fusion, Amp^r	This study
pUTt-pBAD-*eseP*-HA	pUTt derivative containing fragment of L-ara promoter and *eseL*-HA fusion, Amp^r	This study

TABLE S3 Primers used in this study

**Primers**	**Sequence(5' → 3')**
deletion-*eseL*-P1	GAAGATCTAACCAGACTTAGATTAGC
deletion-*eseL*-P2	TAGTTTGTCCGAGCAGGCGGAGAAAGCCG
deletion-*eseL*-P3	GACAAACTAATTCCTTACTG
deletion-*eseL*-P4	ACATGCATGCACCATCGCTGGTTGATGAGG
deletion-*eseM*-P1	GAAGATCTTTTCCACGAGCGACCTAGAGATA
deletion-*eseM*-P2	CCTATAAAACAATATTACCTTCTCAAT
deletion-*eseM*-P3	GTTTTATAGGCTCGCTCTCCTGCG
deletion-*eseM*-P4	ACATGCATGCGTTATATGGCAGGGCGCTTAT
deletion-*eseN*-P1	GAAGATCTGACCCCGATTCCATTTTTCC
deletion-*eseN*-P2	GCCGTCCGCAATCGTTATCTCCATATTGTC
deletion-*eseN*-P3	TGCGGACGGCGGCGTCAGTG
deletion-*eseN*-P4	ACATGCATGCCGGAGGGGTGCAGCAGATCG
deletion-*eseO*-P1	GAAGATCTTCTGCACCGTTTCTGACCAT
deletion-*eseO*-P2	CATCGAGGCGCTTACACCCTCACGTTTTA
deletion-*eseO*-P3	GCCTCGATGCATTGGGATGCCGT
deletion-*eseO*-P4	ACATGCATGCCCAGATACCGATTGCGCCCA
deletion-*eseP*-P1	GAAGATCTCTTCGTTAAACAGCACCAGG
deletion-*eseP*-P2	AGAGGGGGGAATTGGGCTCCTTACTGCG
deletion-*eseP*-P3	TCCCCCCTCTCACCGATAAA
deletion-*eseP*-P4	ACATGCATGCACCAGCGGACGTAAAGGCAA
pBAD-F	ATTGGTTAAAAATTAAGGAGTTATGACAACTTGACGGCTACA
pBAD-R	GGTTAATTCCTCCTGTTAGCCCA
pUTt-*eseL*-F	AGGAGGAATTAACCATGTCTAATAAAATGACTGGC
pUTt-*eseL*-R	ACAGCCAAGCTTTTATTAGGCATAGTCTGGGACGTCATATGGATACA GCGGGGTTACATTAATGGCTG
pUTt-*eseM*-F	AGGAGGAATTAACCATGTCTAATAAAATGACTGG
pUTt-*eseM*-R	CAAAACAGCCAAGCTTTTATTAGGCATAGTCTGGGACGTCATATGGG TTGATAGCAACTACGTTTA
pUTt-*eseN*-F	AGGAGGAATTAACCATGAAAAAAATGAAAAT
pUTt-*eseN*-R	ACAGCCAAGCTTTTATTAGGCATAGTCTGGGACGTCATATGGCTGAA TAACGCCGCAGGC
pUTt-*eseO*-F	AGGAGGAATTAACCATGCGTAAGAGCGTATTGGC
pUTt-*eseO*-R	ACAGCCAAGCTTTTATTAGGCATAGTCTGGGACGTCATATGGACGGT ACTTTTTATGGTA

pUTt-*eseP*-F	AGGAGGAATTAACCATGAATCGTACCTTAACA
pUTt-*eseP*-R	ACAGCCAAGCTTTTATTAGGCATAGTCTGGGACGTCATATGGCTTGC CCACCTGGTGG

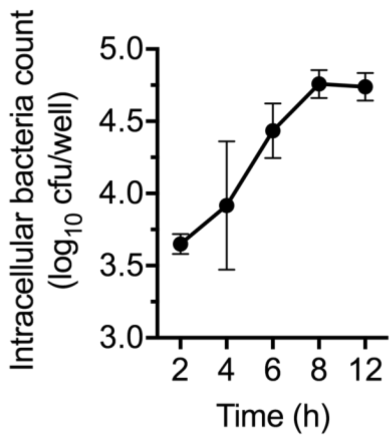

FIGURE S1 Replication of *E. piscicida* in HeLa cells. HeLa cells were infected with *E. piscicida* at a MOI of 100 for 2 hrs, and then treated with 100 μg/ml gentamicin for 1 hrs to kill extracellular bacteria, and after three washes with PBS, incubated in the growth medium containing 10 μg/ml gentamicin for the rest of time. At 2, 4, 6, 8 and 12 hrs post infection, Triton X-100 was added into the cell culture at 1% (v/v) and the lysed mixture was diluted and counted by CFU.

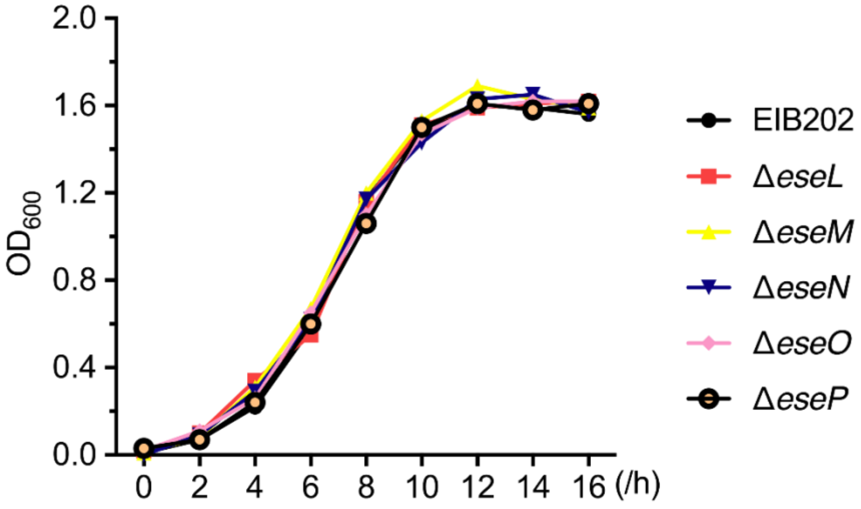

FIGURE S2 Growth of *E. piscicida* wild-type and candidate effector mutants. Strains were cultured in TSB medium with shaking. A 100 ml sample of the bacterial cultures was taken every 2 h to measure the cell density at 600 nm with an ND1000 spectrophotometer (NanoDrop Technologies, Wilmington, Delaware, USA).

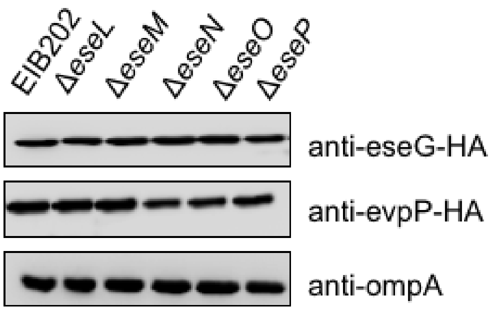

FIGURE S3 T3SS, T6SS and OMV protein secretion in five candidate effector mutants. C-terminal HA-tagged eseG or evpP were expressed in the *E. piscicida* wild-type, Δ*eseL*, Δ*eseM* , Δ*eseN,* Δ*eseO,* Δ*eseP* and their secretion in the supernatant were assessed by Western Blot. The secretion of OMV-dependent protein OmpA in the *E. piscicida* wild-type and five mutants was assessed by using an anti-ompA antibody.

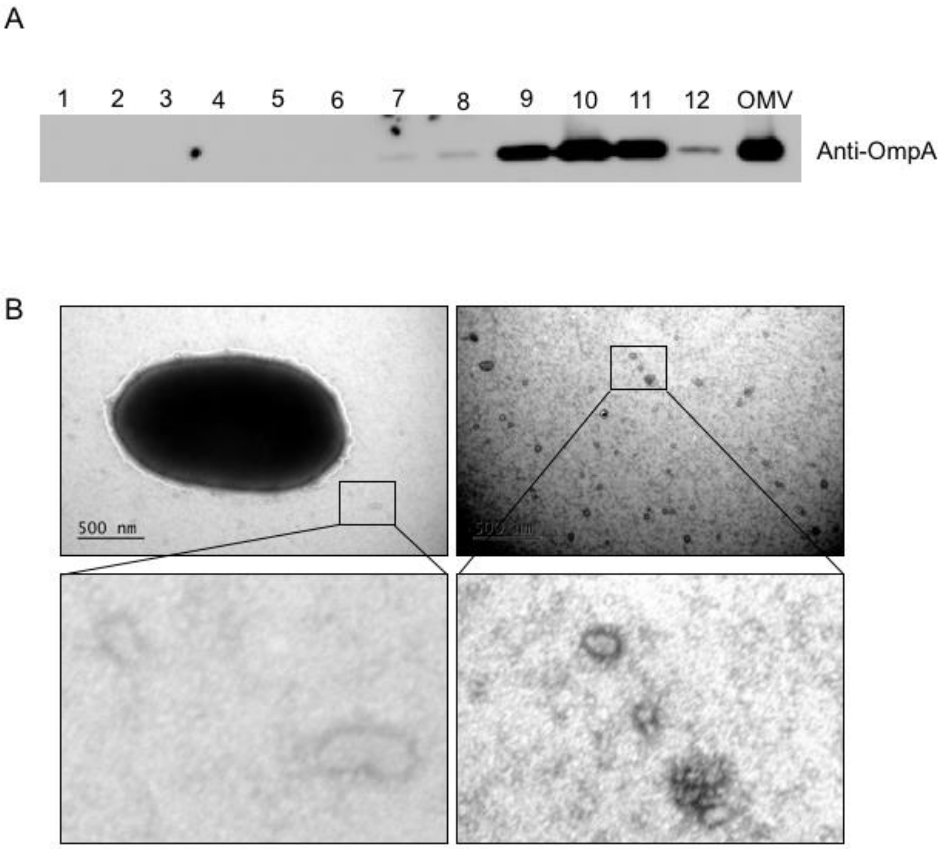

FIGURE S4 OMVs of *E. piscicida*. (A) OMV fractions were prepared as described in Materials and Methods and immunoblotted using anti-OmpA antibody. After adding different OptiPrep/PBS layers, the gradients were centrifuged and twelve fractions of equal volumes (1 ml) were removed sequentially from the top. aliquots (9 µl) of each fractions (lane 1-12, lane 1 is the top fraction, lane 12 is the bottom fraction) and the final OMV fraction (collected from 9-11 fractions) were separated using SDS-PAGE in 12% gels, and analyzed using immunoblotting with anti-OmpA antibody. OmpA is a highly expressed outer membrane protein, which indicates the presence of OMVs. **(B)** *E. piscicida* containing secreted OMVs (left) and purified OMVs (right) are verified by transmission electron microscope. Enlarged images are shown in the below.

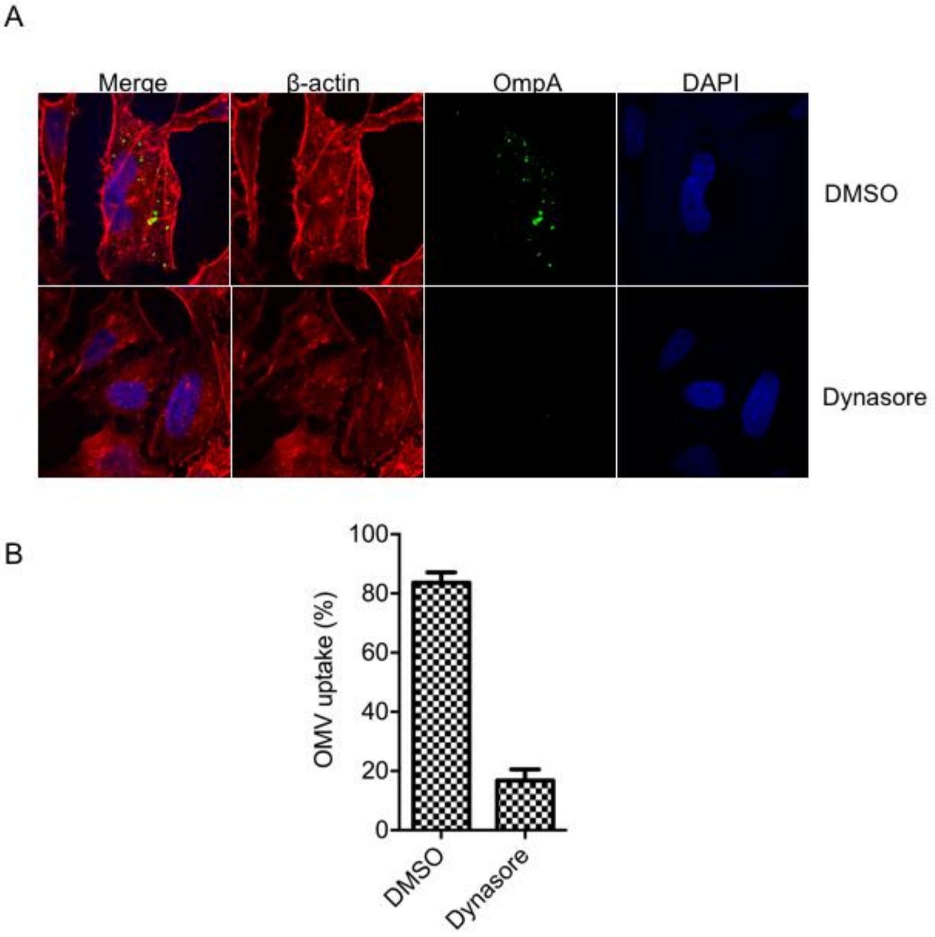

FIGURE S5 OMVs internalization inhibited by dynasore. (A) HeLa cells were pretreated with dynasore (below) or DMSO as a control (upper) for 30 min before infection (80 μM), and the inhibitor was maintained throughout the course of infection. After 7 h infection with wild-type *E. piscicida,* cells were fixed, permeabilized, and immunostained with DAPI, β-actin and OmpA. **(B)** The percentage of ompA-positive cells (% OMV uptake) was determined.
